# Expansion of CD133^+^ colon cancer cultures retaining stem cell properties to enable cancer stem cell target discovery

**DOI:** 10.1038/sj.bjc.6605610

**Published:** 2010-03-23

**Authors:** D D Fang, Y J Kim, C N Lee, S Aggarwal, K McKinnon, D Mesmer, J Norton, C E Birse, T He, S M Ruben, P A Moore

**Affiliations:** 1 Department of Protein Therapeutics, Celera, Inc., 45 West Gude Drive, Rockville, MD 20850, USA

**Keywords:** CSC, CD133, colon cancer, drug target, drug resistance, mass spectrometry

## Abstract

**Background:**

Despite earlier studies demonstrating *in vitro* propagation of solid tumour cancer stem cells (CSCs) as non-adherent tumour spheres, it remains controversial as to whether CSCs can be maintained *in vitro*. Additional validation of the CSC properties of tumour spheres would support their use as CSC models and provide an opportunity to discover additional CSC cell surface markers to aid in CSC detection and potential elimination.

**Methods:**

Primary tumour cells isolated from 13 surgically resected colon tumour specimens were propagated using serum-free CSC-selective conditions. The CSC properties of long-term cultured tumour spheres were established and mass spectrometry-based proteomics performed.

**Results:**

Freshly isolated CD133^+^ colorectal cancer cells gave rise to long-term tumour sphere (or spheroids) cultures maintaining CD133 expression. These spheroid cells were able to self-renew and differentiate into adherent epithelial lineages and recapitulate the phenotype of the original tumour. Relative to their differentiated progeny, tumour spheroid cells were more resistant to the chemotherapeutic irinotecan. Finally, CD44, CD166, CD29, CEACAM5, cadherin 17, and biglycan were identified by mass spectrometry to be enriched in CD133^+^ tumour spheroid cells.

**Conclusion:**

Our data suggest that *ex vivo-*expanded colon CSCs isolated from clinical specimens can be maintained in culture enabling the identification of CSC cell surface-associated proteins.

Increasing evidence suggests that tumour initiation and metastases are dependent on a small sub-population of tumour cells termed cancer stem cells (CSCs) bearing indefinite self-renewal potential and the capacity to differentiate into diverse populations comprising a tumour. Existence of CSCs was first documented in acute myelogenous leukaemia, in which only the CD34^+^/CD38^−^ sub-population of leukaemic cells was shown to proliferate extensively, self-renew, and form new tumours (Bonnet and Dick, 1997). Subsequent studies in solid tumours revealed that CD44^+^/CD24^−^ and CD133^+^ sub-populations contained CSC populations in breast cancer and glioblastoma, respectively (Al-Hajj *et al*, 2003; Singh *et al*, 2004) whereas more recent evidence of their existence has been demonstrated in melanoma (Fang *et al*, 2005; Schatton *et al*, 2008), prostate (Patrawala *et al*, 2006), pancreatic (Li *et al*, 2007), and colon cancer (O’Brien *et al*, 2007; Ricci-Vitiani *et al*, 2007). In addition to tumour initiation, another important attribute of CSCs is their apparent insensitivity to conventional therapies (Bao *et al*, 2006; Liu *et al*, 2006; Phillips *et al*, 2006), hypothesised to contribute to tumour recurrence. Therapeutic targeting of CSC sub-populations therefore represents a novel opportunity to eradicate tumour-initiating, potentially drug-resistant cancer cell sub-populations (Fan *et al*, 2006; Piccirillo *et al*, 2006).

Standard procedures for isolating CSCs from tumour tissues involve cell sorting of a sub-population on the basis of cell surface markers and confirmation of their tumour-initiating activity in xenograft transplantation assays (Clarke *et al*, 2006). In addition, *in vitro* propagation of CSCs applying stem cell culture conditions has been repeatedly reported. Emerging studies have shown that CSC populations isolated and expanded from a variety of tumours, including glioblastomas (Hemmati *et al*, 2003), melanoma (Fang *et al*, 2005), breast (Ponti *et al*, 2005), lung (Eramo *et al*, 2008), ovarian (Bapat *et al*, 2005; Zhang *et al*, 2008), and colon cancers (Ricci-Vitiani *et al*, 2007; Vermeulen *et al*, 2008), frequently grow as non-adherent, three-dimensional (3D) tumour spheres under serum-free conditions. Despite this evidence, it remains contradictory whether *in vitro-*cultured CSCs retain their original phenotype.

Successful cultivation of CSCs *in vitro* not only enables us to study CSCs in a more flexible manner but also provides an additional model in which to test the anti-CSC activity of individual drug candidates or compound libraries through high-throughput screening (Gupta *et al*, 2009). The elucidation of drug resistance in the CSC population has been made on CD133^+^ brain and colon CSCs, largely because of the ability to propagate these cells *in vitro* under a defined serum-free culture condition (Bao *et al*, 2006; Liu *et al*, 2006; Phillips *et al*, 2006). Expansion of CSCs *in vitro* also provides an opportunity to identify cell surface markers to facilitate their detection, enrichment, and potential therapeutic targeting through monoclonal antibody-based strategies. To this end, in this study, we generated and expanded tumour spheres highly enriched in CD133^+^ cells from multiple independent colon cancer specimens under serum-free culture conditions. Upon prolonged expansion, CD133^+^ tumour spheroid cells displayed CSC properties, initiated xenograft tumours, and exhibited resistance to chemotherapy-induced apoptosis. To identify cell surface proteins enriched on cultured tumour spheres, mass spectrometry-based quantitative proteomics was performed on tumour spheres and on the original primary tumour cells from which they were derived. To our knowledge, this is the first demonstration of applying mass spectrometry-based quantitative proteomics to characterise *in vitro-*expanded CSC populations. In addition to confirming the expression of cell surface proteins on CSCs previously identified empirically by flow cytometry, additional cell surface markers not previously associated with colorectal CSCs were also identified.

## Materials and methods

### Tissue process, cell lines, and cell culture

Tissue specimens obtained from Bio-options, Inc (Fullerton, CA, USA) and from the North Shore-Long Island Jewish Health System (Manhasset, NY, USA) were processed within 16 h of surgical removal under appropriate institutional review board approval. Samples were trimmed, sliced, and enzymatically dissociated in a phosphate-buffered saline-based buffer containing dispase (1.5 mg ml^−1^) and DNase (1 mg ml^−1^) for 1 h with agitation at 37°C. A fraction of single primary cells were cultured in non-treated polystyrene cell culture flasks at 37°C, 5% CO_2_ in a humidified atmosphere in a serum-free medium (namely, CSC medium). To generate CSC medium, mouse embryonic fibroblast-conditioned human embryonic stem cell (hESC) medium was mixed with fresh hESC medium at a 1 : 1 ratio and supplemented with 4 ng ml^−1^ bFGF, 10 ng ml^−1^ EGF, 10 *μ*g ml^−1^ bovine insulin (Sigma-Aldrich, St Louis, MO, USA), 5.5 *μ*g ml^−1^ human transferrin (Sigma-Aldrich), and 5 ng ml^−1^ sodium selenite (Sigma-Aldrich; Fang *et al*, 2005). Another fraction of cells was cultured in a serum-containing medium consisting of Dulbecco's modified Eagle's medium supplemented with 20% foetal bovine serum, 0.2 mM non-essential amino acids, 1 mM sodium pyruvate, and 0.075% sodium bicarbonate.

### Cell proliferation and apoptosis assay

Both spheroid and differentiated cells were harvested, disaggregated, plated at 10 000 cells per well in 96-well plates, and cultured in either CSC or serum-containing media, respectively, for 24 h to allow the differentiated population to re-adhere, whereas ⩾90% of the spheroid-derived cell populations remained as single cells in suspension with no evidence of differentiation detected. Cells were then treated with irinotecan (Edwards Medical, Bolingbrook, IL, USA) or vehicle controls. Cell proliferation and apoptosis assays were performed on day 4 using Alamar blue reagent (Invitrogen, Carlsbad, CA, USA) and Apo-One homogeneous caspase-3/7 assay (Promega, Madison, WI, USA), respectively, following the manufacturer's instructions.

### Differentiation and limiting dilution assay

Differentiation was induced by culturing in Dulbecco's modified Eagle's medium supplemented with 20% foetal bovine serum, 0.2 mM non-essential amino acids, 1 mM sodium pyruvate, and 0.075% sodium bicarbonate in flasks coated with 0.3 mg ml^−1^ collagen I (BD Biosciences, San Jose, CA, USA). A limiting dilution assay was performed to evaluate self-renewal capacity. Briefly, spheroid cells were dissociated into single cells and plated in 96-well plates. Final cell dilutions ranged from 5 to 100 cells per well in 0.2 ml of CSC medium. The number of spheres containing more than four cells was counted after 7 days.

### Immunocytochemical staining

Staining was performed as described (Fang *et al*, 2005). Primary antibodies specific for CD133 (Miltenyi Biotech, Auburn, CA, USA), nestin (NES; R&D Systems), musashi-1 (MSI-1; R&D Systems, Minneapolis, MN, USA), and BMI-1 (Upstate, Billerica, MA, USA) were used, with primary antibody binding detected using corresponding Alexa Fluor 488-conjugated secondary antibodies (Invitrogen). The cells were counterstained with 4′,6-diamidino-2-phenylindole.

### Flow cytometry and fluorescence-activated cell sorting

Standard cell surface flow cytometry was used to characterise the samples with phycoerythrin-conjugated CD133 (Miltenyi Biotech), APC-conjugated epithelium-specific antigen EpCAM (CD326, epithelial cell surface antigen, BD Biosciences), and purified E-cadherin (ZyMed, San Francisco, CA, USA) antibodies. Proliferation and cytokeratin expression were measured simultaneously using a fluorescein isothiocyanate 5-bromodeoxyuridine (BrdU) flow kit (BD Biosciences) and anti-cytokeratin antibodies (phycoerythrin-conjugated cytokeratin 7/8 (CAM 5.2), BD Biosciences; or purified cytokeratin 20, Abcam (Cambridge, MA, USA); both worked similarly in our study). Cell sorting was performed on a MoFlo High Speed Cell Sorter (Dako, Carpinteria, CA, USA) using CD133 and EpCAM antibodies.

### RNA isolation and real-time reverse transcriptase PCR

Total RNA was isolated from cultured cells using an RNEasy kit (Qiagen, Valencia, CA, USA), including on-column DNase treatment. Quantitative reverse transcriptase PCR was performed using TaqMan gene expression assays (Applied Biosystems, Framingham, MA, USA) on a Prism 7900HT sequence detection system (Applied Biosystems). Each sample was assayed in triplicate and included a control well without reverse transcriptase. The following genes were tested: connexin 43 (*CX43*) (NM_000165.2; Hs00748445_s1), *BMI-1* (NM_005180.5; Hs00180411_m1), *MSI-1* (NM_002442; Hs00159291_m1), SNAIL1 (NM_005985.2; Hs00195591_m1) *ABCC1* (NM_004996; Hs00219905_m1), *ABCA3* (NM_001089.2; Hs00184543_m1), and *ABCG2* (NP_004818; Hs00184979_m1). Gene expression was quantified relative to 18S rRNA expression, and copy number was estimated assuming 5 × 10^6^ copies of 18S rRNA per cell.

### Anchorage-independent growth assay

Standard soft-agar assays were performed using a cell transformation detection assay kit (Chemicon, Billerica, MA, USA). A 0.8% base agar layer in the serum-containing medium as described above was prepared in six-well culture plates. Viable cells (2500 cells per well) suspended in 0.3% agar diluted in the same medium were layered over the base layer. On the top, spheroid and differentiated cells were provided with CSC and serum-containing media, respectively, and the media were changed twice a week. To evaluate chemosensitivity, irinotecan (5 *μ*M) was included in the feed medium. After 4 weeks, the number of microscopically visible colonies (approximately >100 *μ*m in diameter) was counted.

### Statistical analysis

Cell proliferation and apoptosis data were statistically analysed using two-way analysis of variance to assess the difference in means between spheroid and differentiated cells. If the overall *P*-value was significant (*P*<0.05) between two populations, Tukey's *post hoc* test was used to assess the difference in means for the two group pairs in a given concentration to preserve a low false-positive rate. *P*-value was computed on the basis of studentised range distribution. All analyses were performed using SPlus Windows release 7.0.3 (Statistical Sciences, Seattle, WA, USA). The results shown elsewhere were subjected to a paired *t-*test to determine statistical significance. ^*^P<0.05; ^**^P<0.01.

### Evaluation of tumourigenicity

Tumourigenicity was determined by subcutaneous injection of spheroids into the right flank of five SCID-beige mice (Bioqual, Gaithersburg, MD, USA). Before injection, the spheres were mixed with Matrigel (BD Biosciences) at a 1 : 1 ratio. H&E staining was carried out on paraffin-embedded sections following standard protocols. Tumour volume (*V*) was calculated using the following equation: *V*=*(a*^*2*^ × *b)/*2, where *a* is the width of the tumour (small diameter) and *b* is the length (large diameter), both in millimetres.

### Cell surface protein enrichment and glycopeptide generation

Epithelial cells were enriched up to ∼90% from primary cell populations dissociated from colon tissues through MACS cell separation columns (Miltenyi Biotech) using an anti-EpCAM antibody (BD Biosciences). Cultured tumour spheroid cells were used directly. Dead cells were removed using a dead cell removal kit (Miltenyi Biotech). Viable cells (⩾5 × 10^6^ cells in total) were incubated with 1 mM sodium periodate for 10 min to oxidise carbohydrates of glycoproteins in plasma membrane (Bobbitt, 1956; Schiess *et al*, 2009). After washing with phosphate-buffered saline, cells were lysed and protein concentration was determined by DC assay (Bio-Rad Laboratories, Hercules, CA, USA). Oxidised glycoproteins in the lysate were conjugated to hydrazide resin (Bio-Rad Laboratories) at 4°C overnight with gentle shaking. After removal of non-glycoproteins by thorough washing steps (2 M NaCl, 2% SDS, 200 mM propanol amine (0.1 M NaOAc, pH 5.5), 40% Ethanol, and 80% ethanol), bound proteins were reduced with dithiothreitol and alkylated with ICAT (light reagent, Applied Biosystems). Alkylated proteins were digested with trypsin (37°C overnight). Glycopeptide-bound resins were washed sequentially with 2 M NaCl, 80% ACN/0.1% TFA, 100% MeOH, and 0.1 M NH4HCO3, followed by PNGase F (New England Biolabs, Ipswich, MA, USA) digestion at 37°C overnight to release the glycopeptides.

### LC–MS and LC–MS/MS analysis

Glycopeptides were loaded on a trap column (C18, Grace Vydac 238EV5, 50 mm × 150 *μ*m, 5 *μ*m) and washed for 10 min using 3% buffer B (0.1% formic acid in 90% acetonitrile). Peptides were separated over a C18 monomeric column (Grace Vydac 238EV5, 150 mm × 150 *μ*m, 5 *μ*m) at a flow rate of 1.5 *μ*l min^−1^ using an Agilent 1100 HPLC system (Santa Clara, CA, USA). Peptides were eluted from the column using a gradient, 3–30% buffer B in 215 min, 30–90% buffer B in 30 min. The eluted peptides were analysed using an online QSTAR Pulsar (MDS/Sciex, Toronto, ON, Canada) equipped with an electrospray ionisation source. For liquid chromatography–mass spectrometry (LC–MS) analysis, a 3-s accumulation time was used and data were collected over the mass range of 400–1500 *m*/*z*. For liquid chromatography–mass spectrometry/mass spectrometry (LC–MS/MS) analysis, a 1-s TOF–MS, followed by two 10-s MS/MS scans, was acquired and data were collected over the mass range of 400–1500 *m*/*z* for the TOF-MS scan and 60–2000 *m*/*z* in MS/MS mode. Searches were performed using MASCOT (Matrix Science, Boston, MA, USA) with the Mass Spectrometry protein sequence DataBase (MSDB Imperial College London, London, UK). LC–MS wiff files were transformed to the list of ions using peak detection software ReSpect (PPL, Isleham, UK).

### Proteomic data analysis

For multi-sample analysis, peptide ion peaks of LC–MS maps from individual samples were aligned on the basis of mass to charge ratio (*m/z*), corrected retention time (*R*_t_), and charge state (*z*) as described previously (Kim *et al*, 2009). After intensity normalisation, the list of aligned peptide ions was loaded into Spotfire (Spotfire Inc, Somerville, MA, USA) for differential analysis. Peptides with overexpressed intensity ratios were subjected to LC–MS/MS-based peptide sequencing.

## Results

### Isolation and expansion of CD133^+^ colon cancer-derived tumour spheres displaying capacity for self-renewal and differentiation

**Figure 1:**
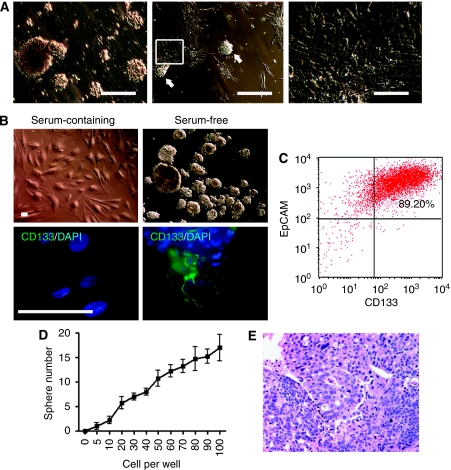
Sphere-forming primary colon tumour cells under stem cell culture conditions retain CD133 expression and tumourigenic potential. (**A**) Formation of non-adherent, three-dimensional (3D) spheroids was observed within 4 weeks in the cultures of primary tumour cells derived from the majority of tumour samples tested (left; bar, 150 *μ*m). In extended culture over a month, some tumour spheroids may become adherent (arrows) and partially differentiated into adherent cell populations (middle; bar, 300 *μ*m). A closeup of the boxed area in the middle panel shows the morphology of epithelial lineages differentiated from a tumour sphere (right; bar, 75 *μ*m). (**B**) Morphology of adherent cells and non-adherent tumour spheres derived from a sorted CD133^+^/EpCAM^+^ population in serum-containing and serum-free media, respectively (top panel). Cell surface staining of CD133 expression (green) was detected by immunofluorescent staining in spheroid populations, whereas their serum-driven adherent counterparts lost the expression of CD133 (bottom panel). Nuclei were counterstained with 4′,6-diamidino-2-phenylindole (DAPI) (blue). Bar, 50 *μ*m. (**C**) Flow cytometry analysis shows that 89.20% of spheroid cells are positive for CD133/EpCAM. (**D**) A fraction of clonally derived spheroid cells reform spheroids in self-renewal assays (mean±s.d., *n*=5). (**E**) Histology of representative xenografted tumours derived from whole-tumour spheres (haematoxylin and eosin (H&E) staining; × 10 objective).

Thirteen independent surgically resected colon tumour specimens of varying pathology stages, together with matched adjacent normal tissue, were dissected to obtain single cell suspensions. Twelve specimens (92%) were derived from primary adenocarcinomas or mucinous adenocarcinomas and one (8%) from a metastatic adenocarcinoma (Table 1). As CD133 has been reported to be a CSC marker for colon cancers, we determined CD133 epithelial cell surface expression levels by flow cytometry. Of 13 tumour samples, 9 comprised of CD133^+^ sub-populations representing >20% of epithelial cells co-staining for EpCAM, with one late-stage tumour (case 12) exhibiting CD133 expression on 85% of the EpCAM^+^ population (Table 1). In contrast, CD133 expression was substantially lower (<5.5%) in the normal adjacent epithelial cells. We next evaluated the growth potential of these primary cells under serum-free conditions as detailed in the Materials and Methods section. As shown in Table 1, sphere-forming characteristics were frequently observed in colon tumour cells (10 of 13) and less in matched adjacent normal tissue cell populations (2 of 13). Sphere formation was observed from both unsorted colon cancer specimens and from those cases (3, 4, 10, and 11) subjected to enrichment by flow cytometry for the CD133-expressing population (Table 1). Approximately 1 in 1 × 10^5^–2.5 × 10^6^ primary tumour cells was capable of forming highly compact spheres (or spheroids). These non-adherent, multi-cellular spheroids usually appeared within 7 days and the majority (∼77%) survived in culture for ∼8 weeks (Figure 1A). During culture, colon tumour spheres frequently became adherent and adopted an epithelial morphology (Figure 1A). To confirm the sphere-forming potential resides within CD133-expressing cells, CD133^+^/EpCAM^+^ cells and CD133^−^/EpCAM^+^ sub-populations were purified from four independent colon tumour specimens (cases 3, 4, 10, and 12). By fluorescence-activated cell sorting, fractionated populations demonstrating purity of 92–95% (CD133^+^/EpCAM^+^) and 94–98% (CD133^−^/EpCAM^+^) were obtained. Within 8 weeks, although all four CD133-enriched populations gave rise to spheroids in the serum-free growth medium, none of the four CD133^−^ populations were capable of generating spheroids (Table 1), indicating that spheroid-producing capability does reside within the CD133-expressing population.

To further characterise colon cancer-derived tumour spheres, effort was made to expand them *in vitro* through prolonged serum-free culture. Three tumour sphere cultures including two (cases 5 and 13) established from unsorted primary tumour cells and another expanded from the CD133^+^/EpCAM^+^ fraction isolated from case 11 were all sustained in culture for more than 5 months. To evaluate more longitudinal changes in the tumour sphere cultures, those derived from case 11 were maintained in culture for more than 22 months, with analysis of BrdU incorporation at months 3, 10, and 15 revealing no change in proliferation rate. Conversely, in a serum-containing Dulbecco's modified Eagle's medium, primary cell populations derived from the same specimen generated adherent cultures with a heterogeneous morphology (Figure 1B; upper left panel). Immunofluoroscence staining shows that tumour spheroid cells, but not adherent cells, expressed colon CSC marker CD133 (Figure 1B; lower panels). These adherent cells underwent senescence within approximately 4–6 weeks. Data presented below are the representative results from the three prolonged spheroid cultures, except for where specified.

Under serum-free, stem cell selective culture conditions, ∼90% of these tumour spheroid cells were positive for CD133/EpCAM, as demonstrated by flow cytometry (Figure 1C). The observation that a fraction of dissociated spheroid cells persistently generated new tumour spheres demonstrates their capacity for self-renewal (Figure 1D).

To investigate whether tumour spheroid cells retain tumourigenic potential upon prolonged passaging, 100 spheres (containing ∼5 × 10^4^ viable cells or 4.5 × 10^4^ CD133^+^ cells) from tumour spheres cultured for over 6 months were injected subcutaneously into the flank of SCID-Beige mice. After 14 weeks, four of five injected mice developed tumours with a mean tumour volume of 336 mm^3^ (±34; s.d.). The H&E-stained section of tumours derived from colon tumour spheroid cells showed fibrous tissues containing adenocarcinoma with foci of necrosis and calcification, histologically identical to the original human colon cancer (Figure 1E and Table 1).

### Differentiation capacity of CD133^+^ tumour spheroid cells derived from clinical specimens

**Figure 2:**
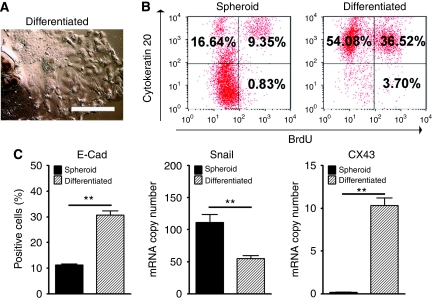
Serum-induced differentiation of clonally derived spheroid cells. (**A**) Under serum-containing media, CD133-expressing tumour spheroid cells became adherent to the substrate and gained epithelial morphology. Bar, 200 *μ*m. (**B**) Flow cytometry analysis shows increased expression of cytokeratin 20 in differentiated, adherent cells (90.60%) compared with its original spheroid population (25.99%). 5-Bromodeoxyuridine (BrdU) proliferation assay reveals that ∼10.18% of spheroid cells incorporated with BrdU compared with 40.22% BrdU incorporation in serum-induced differentiated populations. (**C**) After differentiation, expression of epithelial differentiation markers E-cadherin and CX43 increased along with a decrease in stem cell marker Snail as determined by flow cytometry and real-time reverse transcriptase (RT) PCR, respectively. ^**^P<0.01.

Consistent with observations made with other colon tumour spheres in early cultures, during prolonged propagation, ∼5% of colon tumour spheroid cells spontaneously became adherent and gained epitheloid morphology, indicating differentiation capacity. Meanwhile, single cell-derived clonal populations were obtained from long-term colon tumour spheroid cultures for further characterisation of their differentiation potentials. When removed of growth factors and exposed to 20% serum-containing medium, a significant fraction of clonal cultures (∼70–90%) became adherent. As tumour spheres differentiated, cells migrated out of the adherent spherical colony to form a flat monolayer with typical epitheloid morphology (Figure 2A). After differentiation, the fraction of cells expressing colon epithelium-specific cytokeratin 20 increased significantly (∼90% compared with 26%), whereas BrdU incorporation was observed in ∼40% of differentiated cells compared with ∼10% of undifferentiated tumour spheroid cells (Figure 2B). Increases in the number of cells expressing E-cadherin and elevated *CX43* mRNA levels in the resulting adherent cells confirm differentiation towards epithelial cellular organisation (Figure 2C). A concomitant decrease in the expression of *Snail*, a known transcriptional repressor of E-cadherin and a key regulator of epithelial–mesenchymal transition, was also observed during differentiation (Figure 2C).

### Colon cancer-derived CD133^+^ tumour spheroid cells express a panel of stem cell markers and drug transporter ABCG2 gene

**Figure 3:**
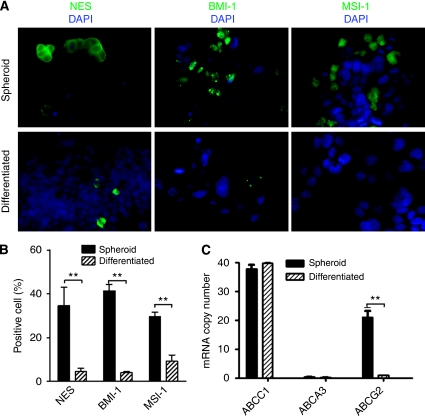
Expression of stem cell markers in colon tumour spheroid cells and their downregulation after differentiation. (**A**) NES, BMI-1, and MSI-1 are detected by immunofluorescence staining in spheroid populations (upper panel). After differentiation, the numbers of cells expressing these stem cell markers reduced significantly (lower panel). (**B**) Decreases in the number of NES-, BMI-1-, and MSI-1-expressing cells after differentiation were quantified by manual cell counting. (**C**) Quantitative reverse transcriptase (RT) PCR studies reveal a decrease in the expression levels of *ABCG2*, but not *ABCC1* and *ABC1* genes, after differentiation. ^**^P<0.01.

The colon cancer derived tumour spheres obtained in this study were evaluated for expression of a panel of stem cell markers, including BMI-1, nestin (NES), and musashi-1 (MSI-1). As shown in Figure 3A, immunofluorescent staining identified ∼30–40% of tumour spheroid cells expressing NES, BMI-1, and MSI-1. Differentiation to an adherent epitheloid phenotype is accompanied by a clear decrease in the percentage of cells expressing NES, BMI-1, and MSI-1 (Figure 3B). Consistent with their stem cell properties, real-time reverse transcriptase PCR analyses demonstrate expression of *ABCC1* and *ABCG2* genes in CD133^+^ colon cancer tumour spheroid cells (Figure 3C). In contrast, no detectable expression of a third member, the *ABCA3* gene, was observed. On differentiation, the level of *ABCG2* mRNA but not of *ABCC1* mRNA is reduced over 10-fold (Figure 3C).

### Colon tumour spheroid cells are more resistant to standard chemotherapy than differentiated progeny

**Figure 4:**
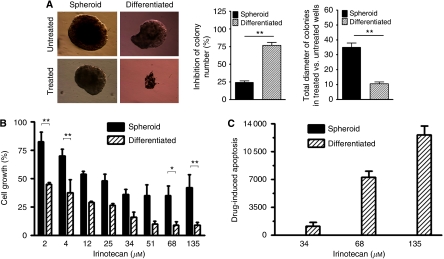
Resistance of colon tumour spheroid cells to standard care chemotherapeutic agent. (**A**) In anchorage-independent assays, after treatment with irinotecan, the number and total sizes of colonies formed by tumour spheroid populations are greater/larger than those formed by adherent populations (left). Untreated spheroid cells were used as baselines to obtain the percentage inhibition (middle) and the relative diameter (right). (**B**, **C**) In culture, tumour spheroid cells are less sensitive to irinotecan-induced growth inhibition and drug-induced apoptosis in comparison with their differentiated adherent counterparts. Apoptosis results are shown as the ratio of the result of treatment with irinotecan over the result of untreated cells as a control, or the ratio of increased caspase-3/7 activity of treatment with irinotecan over the result of untreated cells as a baseline. ^*^P<0.05; ^**^P<0.01.

To determine whether colon tumour spheres display drug resistance, their sensitivity to irinotecan, a component of first- and second-line treatment agents for advanced colon cancer, was first examined in an anchorage-independent growth assay. Compared with counterpart differentiated cell populations, tumour spheroid cells derived from primary tissue display significantly increased resistance to irinotecan-mediated inhibition of anchorage-independent growth as measured both by colony number and size (Figure 4A). Similarly, in cell culture, evaluation of tumour spheres that disaggregated into single cell suspensions demonstrated that they are more resistant, compared with their differentiated progeny, to irinotecan across a wide range of irinotecan concentrations (Figure 4B). Whereas >50% of differentiated cells were growth inhibited by 2 *μ*M irinotecan, 40% of spheroid cells were resistant to growth inhibition at 135 *μ*M, the highest concentration of irinotecan tested (Figure 4B). Resistance to irinotecan-mediated inhibition of cell growth is at least partly contributed by the inability of colon tumour spheres to undergo apoptosis as measured by caspase-3/7 levels (Figure 4C).

### Application of mass spectrometry-based proteomics to identify cell surface-associated proteins expressed on colon tumour spheres

**Figure 5:**
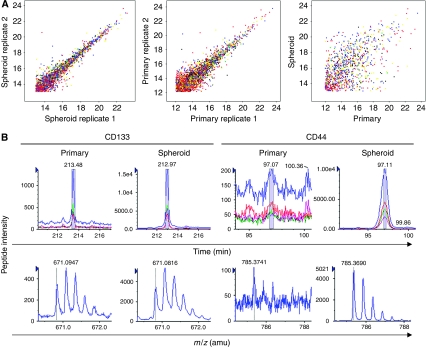
Liquid chromatography–mass spectrometry (LC–MS)-based proteomic analyses reveal expression of additional cell surface markers in CD133^+^ colon tumour spheroid cells. (**A**) Mass spectrometric intensity plots of glycopeptides. Peptide intensities of replicates for both spheroid (left) and primary tumour cell (middle) samples show tight correlation, indicating a similar expression level within the sample. The intensity profile changed dramatically from spheroid to primary cell populations (right), indicating that glycoprotein expression profiles are different in the two populations. Among those differentially expressed proteins, overexpressed proteins (peptides) in spheroid sample (>4-fold) were further sequenced for identification. (**B**) Intensity comparison of glycopeptides from CD133 (LPIQDILSAFSVYVNNTESYIHR) and CD44 (AFNSTLPTMAQMEK) detected in primary *vs* cultured spheroid cell samples. Intensities of these peptides were significantly increased in tumour spheroid cells (11-fold and 45-fold, respectively) as shown in extracted ion current (XIC) chromatograms (upper panel) and corresponding mass spectra (lower panel).

To identify candidate cell surface proteins associated with colon CSCs, a mass spectrometry-based proteomics approach was used. Focus was placed in identifying cell surface proteins preferably enriched in tumourigenic CD133^+^ colon tumour spheroid cells using the original tumour cell population from which tumour spheroid cells were derived as the baseline. A >90% enriched epithelial cell population obtained from the resected colon tumour tissue from patient 11 was processed to facilitate the capture of glycosylated cell surface peptides (details in the Materials and Methods section). Peptides from cell surface were captured in a similar manner from the CD133^+^ tumour spheroid cells expanded from the same patient specimen. LC–MS intensity profiles of the tryptic glycopeptides obtained from each population reveal that ∼5% of the peptides identified show a greater than four-fold difference in their level of expression (Figure 5A). These peptide ions were then subjected to MS/MS sequencing. As anticipated, among the proteins identified to be enriched was CD133. Two glycopeptides from CD133 were identified to be overexpressed in the tumour spheroid population, relative to the primary colon cancer sample (Table 2). Other proteins identified by at least two glycopeptides as overexpressed on tumour spheres include proteins previously associated with colon CSCs: CD44 and CD166. Elevated intensities of CD133 and CD44 proteins in tumour spheroid cells are shown as examples (Figure 5B). Additional cell surface-associated proteins identified as elevated in colon CSCs included CEACAM5, cadherin 17, CD29, and biglycan.

## Discussion

Considering the role of CSCs in driving new tumour formation, spread and recurrence and facilitation of drug resistance, it is of interest to understand their biological properties and design therapeutic strategies to aid their elimination (Dick, 2009). CSCs have been isolated primarily by enrichment using empirically derived cell surface markers, followed by confirmation of their tumourigenesis in immunodeficient mice, such as NOD/SCID mice. For example, in breast cancer, the CD44^+^/CD24^−/low^ sub-population, which comprises ∼11–35% of the total cell population, has been shown to be the population of cells capable of driving tumour formation with as few as 200 cells (Al-Hajj *et al*, 2003). Subsequently, CSC populations in brain (Singh *et al*, 2004), colon (O’Brien *et al*, 2007; Ricci-Vitiani *et al*, 2007), and lung (Eramo *et al*, 2008) cancers have also been demonstrated to reside in the CD133^+^ sub-population, using the xenotransplantation approach. Despite the success of the approach based on cell sorting and *in vivo* propagation of the tumour-initiating population to characterise CSC populations in various tumour types, such an *in vivo* system provides limited material for in-depth molecular and cellular characterisation.

Several key biological properties, including self-renewal and differentiation, are shared by both normal stem cells and CSCs, leading to the term ‘cancer stem cells’ for the tumour-initiating sub-population in human malignancies. Interestingly, although originally designated for *in vitro* propagation of human embryonic and adult stem cells, serum-free culture conditions supplemented with growth factors have been successfully applied to cultivate CSCs derived from solid tumours. Such a growth characteristic was first demonstrated in brain tumours, in which application of a culture condition used to support cultivation of neural stem cells led to the successful isolation of 3D neurospheres bearing CSC properties from human brain tumours (Hemmati *et al*, 2003; Singh *et al*, 2003). Similar tumour spheres enriched for CSC populations have been documented in breast (Ponti *et al*, 2005), melanoma (Fang *et al*, 2005), lung (Eramo *et al*, 2008), ovary (Zhang *et al*, 2008), and colon (Ricci-Vitiani *et al*, 2007; Todaro *et al*, 2007; Vermeulen *et al*, 2008) cancers. Strikingly, in comparison with serum-driven counterparts, cells derived under serum-free culture conditions seem to share more similarities with original tumours, suggesting that they provide a more biologically relevant culture system than that provided by widely used traditional cancer cell lines (Lee *et al*, 2006; De Witt Hamer *et al*, 2008).

In this study, we applied a serum-free culture condition previously proven in both normal and malignant stem cell systems (Fang *et al*, 2005; Yu *et al*, 2006) to cultivate colon CSCs from primary tissues. Tumour sphere formation was observed within 8 weeks for 10 of 13 freshly obtained colorectal tumour specimens processed to obtain viable epithelial cell suspensions. The three specimens from which no tumour spheres were obtained were stage I or stage IIA, raising the possibility that the inability to derive tumour spheres was due to their smaller size and presumably relatively lower CSC content. Interestingly, sphere formation was observed in two normal tissue samples obtained from stage IIIC patients (cases 11 and 12). However, the resulting spheroid cells survived in culture for a relatively shorter period of time compared with their tumour counterparts. Whether these spheres originated from normal colon epithelial stem cells or from invasive and migrating CSCs demands further analyses of the normal adjacent tissue-derived spheroid cells. As controls, adherent primary cultures were also generated under traditional culture conditions using a serum-containing medium. However, the resulting adherent cells lost CD133 expression and exhibited a relatively shorter lifespan compared with spheroid cultures. None proliferated longer than 2 months in our study, indicating that these cells may represent tumour stromal cells or differentiated cancer cells. Our results are in agreement with the earlier study reporting that a serum-containing condition failed to propagate CD133^+^ cells *in vitro* (Ricci-Vitiani *et al*, 2007). In contrast, multiple tumour sphere cultures could be sustained in culture for more than 5 months, including one continually cultured for over 22 months that demonstrated the ability to recapitulate the original tumour phenotype from which it was derived, indicating retention of CSC properties. More detailed *in vivo* studies including limiting dilution studies will be required to determine the percentage of cells within the expanded tumour sphere populations that exhibits CSC properties as performed previously for freshly isolated CD133^+^-enriched populations (O’Brien *et al*, 2007) or CD133^+^ short-term passaged tumour spheres (Ricci-Vitiani *et al*, 2007; Vermeulen *et al*, 2008).

Considering recent reports associating CD133 expression with colon CSCs, flow cytometry analysis was performed to determine the level of CD133 expression on the resected starting specimens. Within the tumour specimens, CD133 expression was observed in 0.26–84.65% of the total cell population, with an average of 30.59% (s.d.: 24.03%), compared with 0.4–5.22% with an average of 2.23% (s.d.: 1.67%) in case-matched normal adjacent tissues. The results of our analyses of CD133 expression in colon cancer are somewhat higher than that reported previously (1.8–25% O’Brien *et al*, 2007; Ricci-Vitiani *et al*, 2007) and more in accordance with those more recently reported by Shmelkov *et al* (2008) in primary human colon tumours. In our approach, we analyse tumour cells immediately after resection by flow cytometry and have also observed high levels of CD133 expression on colon cancer tumour specimens subjected to mass spectrometry-based proteomics (Van Orden *et al*, 2007). Considering the high level of CD133 expression observed on colon cancer tumour cells in some patient samples and the belief that CSCs are only a small sub-population of tumour specimens, our results suggest that CD133 may not be a marker exclusively expressed on functional CSCs. However, the observation that only sorted CD133^+^ tumour cells and not CD133^−^ cells supported tumour sphere formation as shown for cases 3, 4, 10, and 11 indicates that the CSC population does reside within the CD133^+^ cell population (Table 1). Taken together, these observations underscore the need for additional markers to further define the CSC population in colorectal cancer.

The availability of colon tumour spheroid cells derived under serum-free conditions enabled us to further characterise their capability for differentiation. Removal of growth factors and addition of serum facilitate differentiation of colon CD133^+^ CSCs *in vitro* (Ricci-Vitiani *et al*, 2007; Vermeulen *et al*, 2008). In agreement with this finding, we reproducibly observed a differentiation of colon tumour spheres on exposure to serum-containing conditions. The cells became adherent to the substrate of culture flasks rapidly and adapted epithelial morphology. After differentiation, CD133-expressing cells declined from 89.20% in the spheroid populations to 40.5% in the differentiated adherent population (data not shown). The change in CD133 expression was accompanied with increases in proliferation rate and in the fraction of cells expressing E-cadherin and cytokeratin 20. Consistent with the increase in E-cadherin expression, differentiation led to a decrease in Snail, a transcription factor known to repress E-cadherin expression and direct epithelial–mesenchymal transition (Cano *et al*, 2000). The elevated expression of Snail and the decreased expression of E-cadherin in the expanded colon tumour spheres are in line with recent studies indicating that CSCs may be a product of epithelial–mesenchymal transition (Mani *et al*, 2008).

We also assessed additional stem cell and CSC markers, including transcription factor BMI-1, which contributes to proliferative capacity and self-renewal of both normal and malignant stem cells (Lessard and Sauvageau, 2003), and nestin (NES) and musashi-1 (MSI-1), both of which are markers for various stem cell populations, including human colon epithelial stem cells (Nishimura *et al*, 2003). Examination of the expression of stem cell markers NES, BMI-1, and MSI-1 revealed expression in 30–40% of tumour sphere-containing cells compared with <5% of differentiated cells, supporting a view that tumour spheres are not a homogenous cell population but rather a cancer population enriched in CD133 cells retaining CSC properties. Despite the lack of direct evidence for colon cancer, the correlation of elevated CD133 cancer populations in tumour spheres with enriched epithelial–mesenchymal transition and stem cell marker properties is consistent with observations in high-grade brain tumours that elevated CD133 expression correlates with poor prognosis (Beier *et al*, 2008; Rebetz *et al*, 2008).

Drug resistance has been long recognised as one of the major obstacles to effective chemotherapy and radiotherapy of cancer patients. One potential mechanism responsible for drug resistance of cancer cells is the existence of a sub-population of cells within heterogeneous tumours that are inherently resistant to the treatments. Resistance of CSC populations to therapy was first reported in human acute myeloid leukaemia CD34^+^/CD38^−^ stem cells (Costello *et al*, 2000). Since then, resistance to chemotherapy and/or radiotherapy has been linked to CSC sub-populations in various solid tumours, including glioblastoma (Bao *et al*, 2006; Liu *et al*, 2006; Salmaggi *et al*, 2006), breast (Phillips *et al*, 2006), lung (Eramo *et al*, 2008) and colon (Todaro *et al*, 2007) cancers. Consistent with previous studies, our results confirm that colon CSCs exhibit enhanced resistance to the standard chemotherapeutic agent irinotecan compared with their serum-cultured differentiated derivatives. In our drug sensitivity experiments, measurement of cell proliferation has been normalised by vehicle-treated corresponding controls that serve as internal controls. Therefore, the differential response to drug treatment between monolayer (differentiated) and 3D (CSC) cultures seems to result from their intrinsic property, rather than from their physiological differences under distinct culture conditions (2D *vs* 3D). Similar to many other agents in the same class, irinotecan specifically targets rapidly dividing and proliferating cells. Escape of CSC sub-populations from irinotecan-mediated cell toxicity may be attributed to their relatively quiescent proliferating state of cultured CSCs. Furthermore, ATP binding cassette (ABC) drug transporters such as ABCG2 are known to mediate resistance to chemotherapeutics and high levels of ABC drug transporters are suggested to attribute to drug resistance in CSCs (Zhou *et al*, 2008). In our study, we unveil an elevated expression of ABCG2 in colon cancer tumour spheres, which is also anticipated to have a critical role in drug resistance of colon CSCs.

It is of importance that, in addition to facilitating analyses of drug-resistance mechanisms and its applicability in compound screening, the ability to expand CSC-enriched populations *in vitro* provides a sufficient number of cells for analyses by emerging technologies such as mass spectrometry-based proteomics. The proteomic approach directly evaluates global changes in protein expression and identifies cell surface proteins that are overexpressed relative to the original tumour population. Identification of proteins preferably expressed in CSC populations offers novel opportunities to better define and isolate CSCs and potentially provides the basis for the development of targeted therapies to eliminate tumour-initiating cell populations. To this end, we performed proteomic analyses of colon spheroid cells in comparison with the original cell populations isolated from the same patient specimen before expansion as colon cancer tumour spheres. To reduce the complexity of the analyses and to identify changes in proteins that could be potentially amenable to monoclonal antibody-based cell sorting or therapeutic-based strategies, we restricted our focus to the cell surface proteome. Comparison between expanded tumour spheres and the original tumour from which it was identified revealed significant changes in the cell surface peptide expression profile. On the basis of the flow cytometry analysis of the common cancer tumour spheres, MS/MS sequence analyses identified a seven-fold increase in the level of CD133 expression in colon tumour spheres. In addition, overexpression of at least two peptides corresponding to CD166, CD44, CD29, CEACAM5, biglycan, and cadherin 17 was detected in the colon tumour spheres. CD44 has previously been claimed to be a more robust marker for colon CSC isolation by fluorescence-activated cell sorting (Dalerba *et al*, 2007), and our studies here indicated an approximately 40-fold increase in CD44 expression in the tumour sphere culture compared with the expression observed in the epithelial component of the original tumour. CD166 and CD29 expressions have also been reported to be associated with the colon CSC population (Dalerba *et al*, 2007; Vermeulen *et al*, 2008), supporting the CSC-enriched properties of the colon tumour spheres expanded here. In addition, novel cell surface markers associated with CD133^+^ colon CSCs are identified, including CEACAM5, biglycan, and cadherin 17. The roles of these cell membrane proteins in cancer development are well documented. For instance, expression of CEACAMs is associated with poor prognosis in colon cancer (Ishida *et al*, 2004) and they are considered as a therapeutic target (Blumenthal *et al*, 2005). Overexpression of biglycan has been identified in pancreatic cancers (Weber *et al*, 2001). Cadherin 17, also called liver intestine cadherin, is implicated for its role in intestinal cell fate determination, as well as in colon neoplastic development (Hinoi *et al*, 2002). Relatively higher expression levels of these proteins in CD133^+^ colon CSC populations implicate their potential as additional colon CSC markers and as therapeutic targets.

Combined, our data demonstrate that colon CSCs can be isolated and propagated under serum-free, stem cell culture conditions. These tumour spheroid cells retain the expression of well-known cell surface markers, including CD133, CD166, CD44, and EpCAM, as well as other stem cell-associated proteins such as NES, BMI-1, and MSI-1. Colon tumour spheroid cells also exhibit drug resistance to conventional therapy, and possess the capacity for differentiation and tumourigenesis. It is important that colon spheroid CSCs are able to generate tumours that recapitulate the phenotypic heterogeneity and characteristics found in original tumours. Therefore, these cultures resemble colon CSCs identified in freshly isolated tumour specimens and thus provide an *in vitro* tumour model for comprehensive biological analysis of CSC populations, as well as for compound screening. In addition, we identified novel cell surface proteins associated with CD133^+^ colon CSCs, including CEACAM5, biglycan, and cadherin 17.

## Figures and Tables

**Table 1:** Summary of patient population, tumour sample information and sphere-forming capability

**Case**	**Age/sex**	**Tumour stage**	**Tissue type**	**Expression of CD133**^**+**^**/EpCAM**^**+**^ **population (%)**	**Sphere formation**
1	84/F	Stage I	Normal adjacent tissue	2.78	No
		(T2, N0, MX, G3)	Adenocarcinoma	0.26	No
2	92/F	Stage I	Normal adjacent tissue	3.46	No
		(T2, N0, MX, G2)	Adenocarcinoma	4.30	No
3	61/F	Stage I	Normal adjacent tissue	1.00	No
		(T2, N0, MX, G2)	Adenocarcinoma	53.46	Yes^a^
4	88/F	Stage I	Normal adjacent tissue	3.90	No
		(T2, N0, M0, G2)	Adenocarcinoma	23.00	Yes^a^
5	36/F	Stage I	Normal adjacent tissue	0.40	No
		(T2, N0, MX, G2)	Mucinous adenocarcinoma	3.72	Yes
6	76/F	Stage IIA	Normal adjacent tissue	0.62	No
		(T3, N0, MX, G2)	Adenocarcinoma	36.51	No
7	85/F	Stage IIA	Normal adjacent tissue	3.50	N/A
		(T3, N0, M0, G2)	Mucinous adenocarcinoma	29.50	Yes
8	51/F	Stage IIA	Normal adjacent tissue	0.90	No
		(T3, N0, MX, G3)	Adenocarcinoma	4.36	Yes
9	59/M	Stage IIA	Normal adjacent tissue	0.34	No
		(T3, N0, MX, G2)	Adenocarcinoma	45.48	Yes
10	84/F	Stage IIIB	Normal adjacent tissue	5.22	No
		(T3, N1, MX, G2)	Mucinous adenocarcinoma	36.52	Yes^a^
11	62/M	Stage IIIC	Normal adjacent tissue	1.09	Yes
		(T4, N2, MX, G2)	Adenocarcinoma	36.41	Yes^a^
12	70/F	Stage IIIC	Normal adjacent tissue	1.53	Yes
		(T3, N2, MX, G2)	Adenocarcinoma	84.65	Yes
13	38/F	Stage IV	Normal adjacent tissue	4.19	No
		(T4, N2, M1, G2)	Adenocarcinoma (metastasis)	39.49	Yes

Abbreviations: F=female; M=male; N/A=not available (due to contamination).

a Cell sorting performed. Sphere formation was observed only in the CD133^+^/EpCAM^+^ fraction, but not in the CD133^−^/EpCAM^+^ fraction.

**Table 2:** Overexpressed proteins detected by proteomic analyses

**Protein**	**Average ratio of overexpression**^a^ **(s.d.)**	**Peptide detected**
CD166	5.7 (1.6)	TVNSLNVSAISIPEHDEADEISDENR
		IIISPEENVTLTCTAENQLER
		LGDCISEDSYPDGNITWYR
Biglycan	22.1 (2.6)	MIENGSLSFLPTLR
		LLQVVYLHSNNITK
CD44	39.6	AFNSTLPTMAQMEK
Cadherin 17	3.8 (1.2)	APKPVEMVENSTDPHPIK
		KQDTPQYNLTIEVSDKDFK
		GPHFTFSLGSGSLQNDWEVSKINGTHAR
CEACAM5	3.8 (1.3)	LQLSNGNRTLTLFNVTR
		NSGLYTCQANNSASGHSR
		RSDSVILNVLYGPDAPTISPLNTSYR SDSVILNVLYGPDAPTISPLNTSYR TLTLFNVTRNDTASYK
		ITPNNNGTYACFVSNLATGR
CD29	28.4	SAVGTLSANSSNVIQLIIDAYNSLSSEVILENGK
CD133	4.8 (3.8)	LPIQDILSAFSVYVNNTESYIHR
		ILASLDFAQNFITNNTSSVIIEETKK

a Overexpression ratio was calculated using normalised intensities of each peptide detected in spheroid *vs* primary cell (before cultivation) samples.
